# Enhancing systematic literature review adapting ‘double diamond approach’

**DOI:** 10.1016/j.heliyon.2024.e40581

**Published:** 2024-11-20

**Authors:** Hari Prasad Pandey, Tek Narayan Maraseni, Armando A. Apan

**Affiliations:** a Department of Forests and Soil Conservation, Ministry of Forests and Environment, Kathmandu, Nepal; b Centre for Sustainable Agricultural Systems (CSAS), University of Southern Queensland, Toowoomba, Queensland, 4350, Australia; c Institute of Life Sciences and the Environment (ILSE), University of Southern Queensland, Toowoomba, Queensland, 4350, Australia; d Northwest Institute of Eco-Environment and Resources, Chinese Academy of Sciences, Lanzhou, 730000, China; e School of Surveying and Built Environment, University of Southern Queensland, Toowoomba, Queensland, 4350, Australia; f Institute of Environmental Science and Meteorology, University of the Philippines Diliman, Quezon City, 1101, Philippines

**Keywords:** Literature searching, Research bias, Two-stage process, Systematic review

## Abstract

Current literature review frameworks often lack quantification of literature volume, guidance on keyword inclusion, confidence in identifying knowledge gaps, and the formulation of research questions, which can lead to potential bias in research. This undermines the validity and reliability of reviews. In this paper, we aim to enhance the systematic literature review (SLR) methods—starting with a review of review literature followed by a review of empirical literature—to address these challenges. By critically evaluating existing SLR approaches, we propose an enhanced SLR method, termed 'double-stage SLR,' designed to identify gaps, define research questions, develop strategies to overcome challenges, and deliver high-quality review work. This approach mirrors the Double Diamond Approach (DDA), which we refer to as ‘DDA in SLR’, viewing systematic reviews as projects aimed at addressing existing issues in review processes. The DDA in SLR involves a two-stage process: first, reviewing existing reviews to formulate research questions, and then examining empirical research to meet review objectives. This method does not challenge existing SLR frameworks but rather acknowledges them as the foundation for enhancement by systematically reviewing both review and empirical literature, ultimately optimizing review quality. Additionally, this enhanced method reduces risks and bias in SLR, as validated through practical application, and encourages its use to produce high-quality outputs in research, academia, and beyond.

## Highlights

• Existing literature review frameworks lack in guidance on quantifying background information and searching for keywords.
• We critically reviewed the existing systematic literature review (SLR) methods and identified key limitations in them.
• We propose a method called the ‘Double Diamond Approach (DDA)’ in SLR to address the prevailing issues associated with SLR.
• DDA in SLR involves a two-cycle SLR: a review of reviews and a review of empirical studies.
• DDA in SLR reduces risks, minimizes leftover chances and bias in search strategies, and leads to quality review outcomes.

## Graphical abstract

**Figure:**
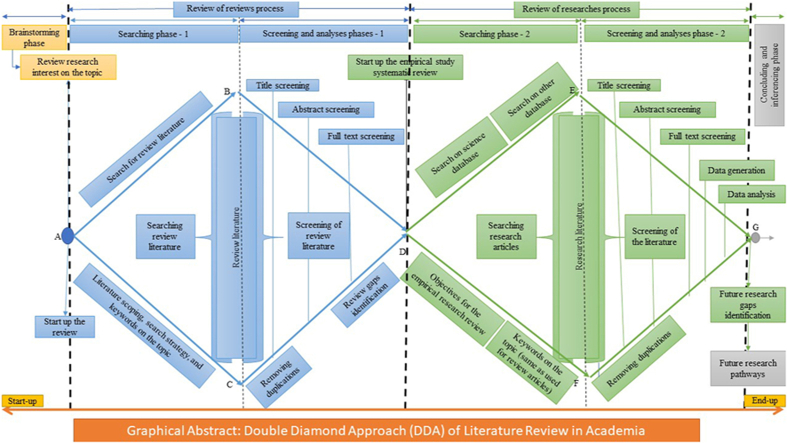

## 1 Introduction

The literature review is a critical evaluation and synthesis process in academia, which ensures the best use of existing research for further advancement of knowledge and policy decisions [1,2]. This facilitates summarizing previous research, allowing identifying the research gaps, and providing future direction based on the existing body of knowledge [1,3]. It has also been used in depicting research trends, establishing the theoretical framework, developing research questions, and the systematic evaluation of cause-and-effect relationships in the area of interest [[4], [5], [6], [7]]. Recognizing its widespread application, literature review is becoming a primary task for exploring broad knowledge in a given field and identifying gaps in past research to develop a review project [8,9]. To achieve this, it is believed that the literature review method should be comprehensive, rigorous, replicable, smart, and transparent while considering the specificity and sensitivity of the knowledge [1,7]. For this reason, numerous approaches have been adopted for the literature review so far. One of them is the systematic literature review (SLR).

SLR has become a widely adopted literature review approach in recent years due to its methodological rigor, which ensures the validity, reliability, and repeatability of the review process [1,5,10,11]. Scholars regard it as a rigorous and comprehensive method for identifying, analyzing, and synthesizing existing scientific knowledge related to a specific research question or topic [5,12,13]. However, while performing SLR, scholars face some sorts of difficulties that the outcomes become more convincing than the more informal approach to generating background information commonly encountered in many research papers, PhD theses, and higher education studies [14,15].

The SLR process involves several steps, including defining the research question, searching for relevant studies, screening and selecting studies based on predetermined criteria, extracting data, analyzing and synthesizing the information, and reporting the findings [[6], [7], [8],^13^,^15^,^16^]. In academia, it is often used to identify knowledge gaps for further research or review work [17,18]. Identifying a major gap in the existing body of knowledge is a milestone for proceeding with a review work. This is not only true in academic research projects but also in other development fields elsewhere (e.g., industry and corporations) to design a multi-year research project or write a grant for a developing project [6,19,20]. Further, SLR plays a crucial role for policymakers, clinicians, and other professionals who need to make evidence-based decisions [21,22]. Therefore, the review method should be smart and trustworthy for quality outcomes. However, the existing practice of reviewing the literature solely depends on the discretion of the researchers utilizing the body of literature and knowledge under their domain and preference [23], which poses a significant bias in literature selection, in turn, entirely to a review quality [9,24].

This paper aims to critically analyze the prevailing SLR methods in research and academia to enhance the review process and reduce researchers’ selection bias, thereby ensuring higher-quality research outcomes. Specifically, this study examines contemporary SLR protocols, identifies critical gaps in the review process, and proposes a double-step SLR process to reduce bias in literature searches, background information, and keyword selection in the field of interest. To achieve this, we systematically review existing methodological and selected review articles, critically analyze them, and propose enhanced methods for future SLR work. It is important to note that this study and the proposed enhanced method do not challenge existing SLR frameworks but rather build upon them as a foundation for improvement by systematically reviewing both review and empirical literature, thereby optimizing review quality. Additionally, this enhanced method reduces risks and biases in SLR, validated through practical application, and encourages its use for producing high-quality outputs in research, academia, and beyond.

## 2 Major prevailing approaches to systematic literature review

Various approaches and methodological frameworks have been developed globally for systematic literature reviews (SLR). For example, the Preferred Reporting Items for Systematic Reviews and Meta-Analysis (PRISMA) protocol are widely used in SLR studies [6,13,25]. PRISMA provides a general framework that guides the development of systematic review protocols. It outlines what is already known in a field of interest and provides a robust checklist and reporting protocol for systematic reviews [26]. However, this framework does not offer guidance on selecting background literature, finalizing keyword searches, identifying research gaps, determining how much background literature is sufficient to formulate research questions, or informing the preliminary information needed before starting the systematic review.

Similarly, the Collaboration for Environmental Evidence [7] approach presents another methodology for systematic literature review (SLR). This framework outlines the various steps required to complete the SLR process, from problem identification to communication and outreach [24]. In line with this, Alexander (2020) [1] further elaborates on the SLR methodology concerning quality standards. However, it remains unclear how to quantify the literature to formulate research questions (or identify review research gaps) and to develop an effective search strategy while conducting the SLR.

Likewise, the International Collaboration for Automation of Systematic Reviews (ICASR) [16] approach addresses the problem of duplication of efforts and provides guidelines for validating the currently available tools and techniques in the field of systematic literature review (SLR). Moreover, subject-specific review methods have also been suggested, recommending an approach that starts with defining the research question, followed by scoping the review and conducting a search for relevant papers [14,27]. However, these approaches do not specify the volume of literature that should be searched, how much literature is sufficient to identify research gaps, or how to formulate research questions from the existing reviews in the field of interest. Consequently, researchers often rely on limited literature in their domain for background information before conducting a review [28]. This, in turn, introduces biases in identifying review research gaps using restricted literature, leading to leftover bias [12,29,30], diminishing credibility [18], raising ethical concerns [31], and resulting in significant problems with the reproducibility of scientific analysis and synthesis [32]. In summary, all the major prevailing approaches to literature reviews are debatable [28].

**Fig. 1:**
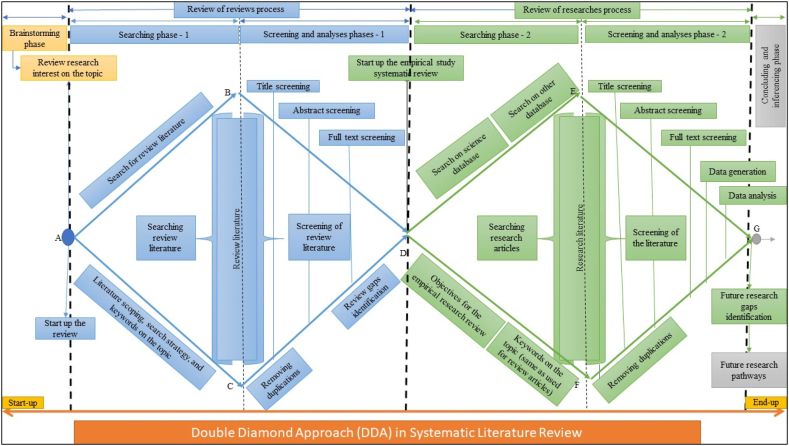
The proposed framework of the Double Diamond Approach (DDA) for SLR in academia and scientific diaspora (Adopted by the UK Design Council, 2005 [33]). Alphabets (A–G) are arbitrarily assigned to denote the corner points of the diagram to facilitate the understanding of this approach and steps. The text has no scaling but just indicates the steps and extent the entire frame.

In this context, there is a need to develop a comparative understanding of systematic literature review (SLR) to enhance credibility, reduce bias, and improve transparency and replicability. To achieve this, we propose the two-step, double-stage, or Double Diamond Approach (DDA) in SLR. DDA is a widely recognized framework in design thinking, developed by the UK Design Council [33], and provides a structured process for problem-solving and innovation [34]. Given that review work is a form of project, it should follow the four DDA phases: discover, define, develop, and deliver [35]. We have termed this the 'DDA in SLR' approach due to its resemblance to the DDA framework. In the context of a review, the discovery phase involves understanding the review problem by gathering insights and exploring the existing body of knowledge. In the second phase, it is essential to define the objectives and research questions. The third phase focuses on developing a protocol to address the research gaps identified in the review, and the final phase involves delivering the outcomes and/or future directions. In this 'DDA in SLR' framework, the first diamond focuses on the review of review literature, while the second diamond addresses the review of the empirical literature, as illustrated in Fig. 1. This approach mirrors the DDA, treating systematic reviews as projects designed to address challenges in the review process. This innovative approach is expected to benefit not only the educational and scientific fields but also policymaking in education, science, and research globally, ensuring high-quality review outcomes.

## 3 Method

We conducted two stages of systematic literature review (SLR), referring to this process as the “DDA in SLR” to enhance the literature review process. First, we used the following search strings: [(“Literature review” OR “Systematic literature review” OR “Systematic review” OR “Literature search” OR “Scientific literature”) AND (“Protocol” OR “Strategy” OR “Procedure” OR “Guideline” OR “Step” OR “Critique” OR “Method” OR “Approach” OR “Matrix” OR “Framework”)] in Web of Science and Scopus to explore the extensive review literature, specifying “review articles”. The reason for choosing these scientific databases was to extract high-quality peer-reviewed review articles to explore consolidated scholarly work from these largest science databases. However, the search results yielded over 0.3 million review articles containing these keywords. This number is overwhelming, making it impractical to review each article. Also, using the field code function would introduce bias in the selection process that we did not follow.

Next, we refined our search keywords to: [(“Literature review” OR “Systematic literature review” OR “Systematic review” OR “Literature search” OR “Scientific literature”) AND (“double step” OR “two-step” OR “double cycle” OR “double diamond”)] in these databases to narrow down the literature output for our intended objectives. From this revised search strategy, we identified 1163 articles in Scopus and 1131 articles in Web of Science. Additionally, we conducted searches in Google Scholar, and Google and performed index and reference searches to triangulate any missing literature. We screened all these articles at the title level in the first stage, at the abstract level in the second stage, and at the full-text level in the final stage using the CADIMA online platform [36].

We found that no methodologies specifying “DDA”, “double stage” or “two-step” for systematic literature review (SLR) have been developed in academia from these databases. Additionally, we screened review articles using the keywords “double step”, “two steps”, “double cycle”, or “double diamond” in the topic level at all mentioned databases. This search yielded references to the terms: Double Diamond Design Approach [34,35,37] and two-step technique [[38], [39], [40]] for a few articles. We then skimmed the full texts of these articles; however, they did not focus on enhancing the systematic review process using these techniques but rather on identifying problems and prospects within their respective fields of interest using these procedures [14]. Based on these findings, we confirm that the DDA has not been used as an approach for SLR in academia or the broader scientific community to date.

## 4 Proposed framework: DDA in SLR

The Double Diamond Design Approach (DDA) was initially developed by the UK Design Council in 2005 to identify and solve problems, particularly in the corporate world and private companies [20,33]. Scholars have also emphasized the applicability of the DDA approach to any type of project by customizing it [41,42]. For example, this approach has been utilized in various fields, including human health-related issues [26,34], sustainability in supply chain management practices [37], and developing strategies for environmental sustainability [35]. It is regarded as a competitive model for identifying problems and overcoming them [43]. Considering SLR as a ‘project’, we propose a DDA in conjunction with an SLR framework for the education and scientific community (Fig. 1).

DDA in SLR begins with the brainstorming phase and concludes with the inference of future pathways based on the findings. This process includes but is not limited to, formulating courses of action, making policy decisions, developing research questions for new projects, and identifying knowledge gaps for future research. The first diamond represents the review of the reviewed literature, while the second pertains to the review of empirical research literature (Fig. 1). Axes AB and AC form the acute angle ∠BAC, with the angle's size depending on the breadth of the literature search and its availability in the relevant field. In contrast, axes BD and CD create an acute angle at point D, reflecting the volume of the review literature spanning from B to C. At point D, researchers can identify the review gaps for further investigation.

Similarly, axes DE and DF form an acute angle at D, leading into the empirical research literature review diamond. The size of this angle depends on the volume of research literature available in the field of interest. Meanwhile, axes EG and FG determine the size of the acute angle at G within the second diamond, indicating the number of future pathways addressing the research questions formulated at point D, which marks the end of the first diamond and the start of the second. In Fig. 1, we have named the pathways from A to G: A – ACTION and G – GO, thus creating the “ACTION to GO Model” of SLR in academia using DDA. This naming facilitates easy communication and understanding of the entire framework.

At the outset, scholars should brainstorm to develop a preliminary concept for their topic of interest and generate a list of keywords for the background literature search by consulting a dictionary for synonyms. These synonyms will aid in exploring relevant review literature in the initial stages. At point A (Fig. 1), the researcher begins by searching with the keywords generated from brainstorming and synonym exploration. At this stage, it is beneficial to locate typological review literature to ensure that all relevant typologies in the field are covered, which can be refined through iterative screening and skimming in a back-and-forth process.

The next step, after finalizing keywords for the review literature, is to run searches in databases using these keywords and the scope of the review research as outlined in the SLR (e.g., Refs. [1,7]). As review literature accumulates between points B and C, duplicates should be removed manually using tools such as Excel, EndNote, Mendeley, Zotero, or specialized review management software like EPPI-Reviewer, RevMan, CADIMA, Covidence, etc. Before entering the screening stage, scholars need to finalize the search keywords used in prior review literature in the field. These keywords will be used again in the next stage when searching empirical research literature. Subsequently, screening of the review literature is conducted as needed (title, abstract, keywords, full text) to identify authentic knowledge gaps in the discipline at point D. A summary table categorizing (sub)themes covered or lacking in past reviews can help pinpoint unconsolidated information and themes—these gaps indicate areas for further review work. Meanwhile, these review gaps inform the research questions for the empirical literature review in the second phase. Additionally, as scholars progress to point D, they can refine search strings (keywords) as they review the literature before moving on to the second phase—the research review cycle. Furthermore, literature on empirical studies misclassified in the review category by online databases can be reserved for use in the next review phase.

Similarly, using the previously identified search strategies and keywords, the search can be narrowed to empirical research literature within the field of interest across various databases and grey literature. This empirical literature will then be screened, with duplicates removed according to predefined objectives. If the search yields some review literature miscategorized as empirical, this can help refine search keywords, uncover new insights, and reduce any remaining bias. After reviewing the research literature at point G, research gaps are identified, and the implications of the review outcomes for future directions can be outlined. The second stage of reviewing empirical literature based on identified research gaps has been common in past methodologies [6,7,13,14,16,27]. However, our proposed method enhances this by including a ‘review of reviews’, beginning with brainstorming and proceeding systematically to identify knowledge gaps in review articles and finalizing the searching keywords by exploring all the review literature in the field of interest those used in the past scholarly works. Additionally, empirical research literature miscategorized under reviews is reserved for inclusion in the empirical review process and vice versa. Through this double-cycle review process, the quality of review outcomes is expected to be enhanced, and existing review bias can be diminished.

## 5 Significance and limitation

We validated the double-stage SLR approach using prior scholarly review work. Scholars have emphasized the benefits of adopting a double-stage review process for mapping findings from earlier studies and identifying research gaps [44]. This research also highlighted the advantages of categorizing themes in review articles. Additionally, conducting reviews of empirical research can address gaps identified during the review of reviews, such as publication trends, coverage, nature, and thematic focus of past scholarly work [44]. Adopting double-step review approach helps identify research voids and consolidates information for decision-making through high-quality review output [45,46]. Initial experiences revealed that our proposed approach offers three main advantages compared to existing literature review methods (one-step SLR) [[44], [47]]. These are: 1) It allows for the inclusion of research literature listed under the 'review category' that may belong to the 'research category,' and vice versa. This adherence to the principle of 'no stone unturned' minimizes the chance of missing keywords and key literature in the field of interest and reduces leftover bias in literature searching. 2) It assists in refining search keywords through an iterative process used in previous review studies before conducting the main literature search for empirical articles. This means that the reviewed literature offers ample scope and strengthens the existing typologies to confirm the extent and relevance of the search keywords in the field of interest. 3) It provides reviewers with the confidence to assert that their 'review project' is unique and novel among the existing scholarly works, as both sets (reviewed and empirical) of literature are assessed and incorporated through this approach [24,44]. Ultimately, this will help reduce scholars’ hesitancy to claim that there is 'scarcity in the existing knowledge' and 'to the best of our knowledge,' thereby allowing them to present their scholarly review work as unique and novel within the frontiers of existing bodies of knowledge in the discipline [47].

Building on the merits of the DDA in SLR, this strategy reduces risks, ensures specificity and sensitivity, and minimizes bias in the overall literature review projects [5]. Specifically, this approach addresses bias throughout the review process—including scoping, identifying research gaps, formulating research questions, typological selection, literature search, and generating inferences—while increasing credibility, resolving ethical risks, enhancing validity and confidence in the research, and providing a comprehensive view of reproducibility in scientific analyses and syntheses. However, this approach requires more time and effort for completion. Additionally, this paper relies solely on an online search strategy limited to a few science databases (Web of Sciences, Scopus, and Google Scholar), published in English, which may overlook certain types of literature that are not available online, are non-English, or fall outside the selected databases. Books and book chapters are also beyond the review scope of this paper. Nevertheless, this novel approach can be applied not only in education and research [1,3,17,32] but also by decision-makers in education and research, and beyond [2,21,29] as a reference for further enhancements in systematic review work. Readers should note that the DDA in SLR does not aim to replace existing SLR methods but rather enhances them by adopting double steps in literature searching and assessment to ensure comprehensiveness and high-quality outcomes. Therefore, we highly recommend adopting the DDA in SLR to improve the quality of review outputs in the future.

## 6 Conclusion

Improvements in the literature review process are ongoing, with systematic literature review (SLR) emerging as a prominent method in contemporary research. However, existing SLR approaches face criticism. We systematically reviewed the methodological literature adopting systematic review and identified the critical gaps. To overcome such existing gaps in the methods, we introduce the Double Diamond Approach (DDA) in SLR aims to address these shortcomings and advance the field of review works. Acknowledging existing SLR methods and building on these, this approach introduces a complete, two-step review cycle by systematically reviewing the review literature before beginning the empirical literature review. This double-step SLR strategy (DDA in SLR) seeks to mitigate biases and ethical concerns while increasing confidence, efficiency, and transparency, and it has been validated through multiple review projects. By providing a comprehensive framework, the DDA in SLR enhances the quality and credibility of reviews, fostering growth in the discipline. We advocate for the adoption of DDA in SLR to achieve superior review outcomes and strengthen the integrity of scholarly work.

## CRediT authorship contribution statement

**Hari Prasad Pandey:** Writing – review & editing, Writing – original draft, Visualization, Validation, Methodology, Formal analysis, Data curation, Conceptualization. **Tek Narayan Maraseni:** Writing – review & editing, Validation, Supervision, Software, Methodology, Conceptualization. **Armando A. Apan:** Writing – review & editing, Validation, Supervision.

## Data availability

No specific data have been used for this study.

## Funding declaration

This research does not receive specific funding to carry out.

## Declaration of competing interest

The authors declare that they have no known competing financial interests or personal relationships that could have appeared to influence the work reported in this paper.
